# The Evolution of Temperature and Desiccation-Related Protein Families in Tardigrada Reveals a Complex Acquisition of Extremotolerance

**DOI:** 10.1093/gbe/evad217

**Published:** 2023-11-29

**Authors:** James F Fleming, Davide Pisani, Kazuharu Arakawa

**Affiliations:** Institute for Advanced Biosciences, Keio University, Tsuruoka City, Yamagata, Japan; Natural History Museum, University of Oslo, Oslo, Norway; Palaeobiology Research Group, School of Biological Sciences and School of Earth Sciences, University of Bristol, Bristol, United Kingdom; Institute for Advanced Biosciences, Keio University, Tsuruoka City, Yamagata, Japan

**Keywords:** Tardigrada, phylogenetics, protein evolution, cryptobiosis

## Abstract

Tardigrada is an ecdysozoan lineage famed for its resilience. Tardigrades can tolerate high doses of radiation, low-oxygen environments, desiccation, and both high and low temperatures under a dormant state called “anhydrobiosis”, which is a reversible halt of metabolism upon almost complete desiccation. A large amount of research has focused on the genetic pathways related to these capabilities, and a number of genes have been identified and linked to the extremotolerant response of tardigrades. However, the history of these genes is unclear, and the origins and history of extremotolerant genes within Tardigrada remain a mystery. Here, we generate the first phylogenies of six separate protein families linked with desiccation and radiation tolerance in Tardigrada: cytosolic abundant heat-soluble protein, mitochondrial abundant heat-soluble protein, secretory abundant heat-soluble protein, meiotic recombination 11 homolog, and the newly discovered *Echiniscus testudo* abundant heat-soluble proteins (alpha and beta). The high number of independent gene duplications found amongst the six gene families studied suggests that tardigrades have a complex history with numerous independent adaptations to cope with aridity within the limnoterrestrial environment. Our results suggest that tardigrades likely transitioned from a marine environment to a limnoterrestrial environment only twice, once in stem Eutardigrada and once in Heterotardigrada, which explains the unique adaptations to anhydrobiosis present in both classes.

**Significance**

The ability of tardigrades to resist desiccation (anhydrobiosis) has been the focus of a great deal of research in recent years. However, the phylogenetic relationships of the proteins that drive this behavior are yet unknown. Here, we present the first phylogenetic analysis of cytosolic abundant heat-soluble protein, mitochondrial abundant heat-soluble protein, and secretory abundant heat-soluble proteins, alongside the recently discovered *Echiniscus testudo* abundant heat-soluble alpha and beta proteins, and use them to start understanding the evolutionary history of tardigrade anhydrobiosis.

## Introduction

Tardigrades, also known as water bears, have attracted research interest in recent years for their extremotolerant traits (Barnes 1987; Møbjerg et al. 2011; Yamaguchi et al. 2012; Hashimoto et al. 2016). These meioscopic animals have colonized every continent on earth, including Antarctica, and species are present across freshwater, marine, and terrestrial biomes (Nelson et al. 2010). Tardigrade terrestrialization appears to have occurred multiple times within the phylum, as multiple, independent clades of terrestrial Tardigrada exist (Guil et al. 2018; Fleming and Arakawa 2021).

Tardigrada can be split into two or three classes—Eutardigrada, Heterotardigrada, and the dubious, monotypic Mesotardigrada (Grothman et al. 2017). The Eutardigrada are defined by their claws and buccopharyngeal apparatus, whereas the Heterotardigrada are morphologically diverse and classified according to their cuticular extensions, dorsal plate patternings, and cephalic appendages, as well as using their claws and buccopharyngeal apparatus (Barnes 1987; Nelson et al. 2010). The Heterotardigrada possess lateral appendages or “thorns,” which are not found in Eutardigrada (with the exception of some species within the Calohypsibiidae; Michalczyk and Kaczmarek 2005), which have been proposed to serve as additional sensory apparatuses. The lack of lateral appendages easily differentiates Eutardigrada even from the morphologically less adorned members of the Heterotardigrada (Hansen et al. 2003).

Tardigrada are capable of assuming many forms of extremotolerance, with species variably showing an ability to resist drought, heat, cold, pressure, radiation, and even vacuum (Møbjerg et al. 2011). One particular form of extremotolerance is anhydrobiosis, which is the ability to survive drought (Arakawa 2022). Tardigrades accomplish this by entering a cryptobiotic state known as “tun.” Three tardigrade-specific protein families associated with anhydrobiosis were initially identified—cytosolic abundant heat-soluble (CAHS) protein, mitochondrial abundant heat-soluble (MAHS) protein, and secretory abundant heat-soluble (SAHS) protein (Yamaguchi et al. 2012; Fukuda et al. 2017; Fukuda and Inoue 2018). Some tardigrades possess multiple copies of the genes that code for these proteins, but the true taxonomic breadth and internal diversity of these families is poorly understood, with much of our understanding coming from research in two closely related eutardigrade species: *Hypsibius exemplaris* and *Ramazzottius varieornatus* (Yamaguchi et al. 2012; Fukuda et al. 2017; Fukuda and Inoue 2018; Arakawa 2022). Some heterotardigrades appear to possess a variant cryptobiotic pathway using two families of abundant heat-soluble proteins first identified in *Echiniscus testudo*, and usually referred to as *E. testudo* abundant heat-soluble (EtAHS; Murai et al. 2021) proteins. However, due to poor sampling, it is not clear whether EtAHS is unique to *Echiniscus*, the Echinisciidae, Heterotardigrada, or even more broadly distributed across Tardigrada (Murai et al. 2021).

**Fig. 1.**
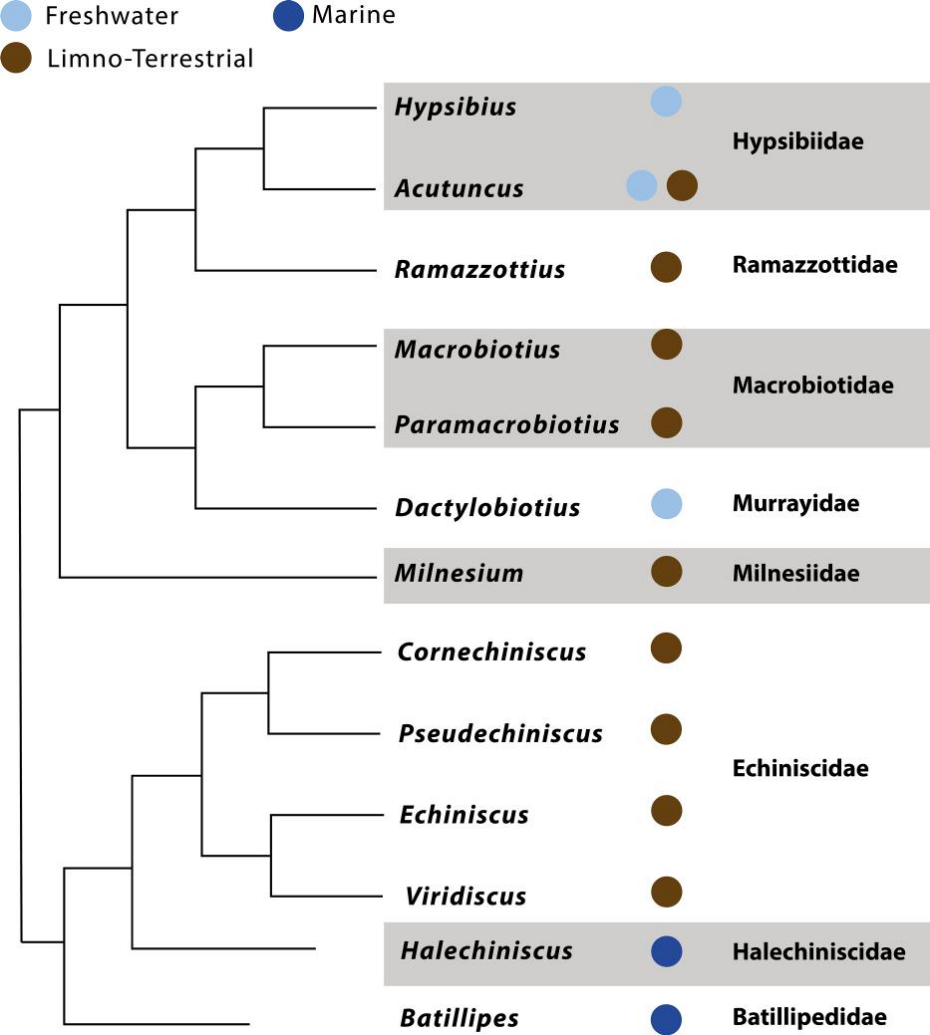
A cladogram depicting the life habits of the tardigrades sampled in this study. The habitat of each sampled tardigrade genus is indicated as circles on the leaves: marine environments are in dark blue, freshwater in light blue, and limnoterrestrial in brown.

In addition to tardigrade-specific tolerance proteins, tardigrades also possess stress resistance proteins found across Metazoa more broadly, such as meiotic recombination 11 (MRE11), which has been implicated in desiccation tolerance in other ecdysozoans (Mahroof et al. 2005) and in vertebrates (Fangue et al. 2006). However, although MRE11 is usually found in a single copy, some tardigrades seem to possess multiple MRE11 copies—with the taxonomic breadth of these duplications being still unknown. It has been suggested that duplications in these protein-coding families relate to increased desiccative tolerance (Fangue et al. 2006; Hashimoto et al. 2016). We hypothesize that heightened resistance to desiccation in living tardigrades emerged as an adaptation to environments with a higher risk of desiccation, that is, terrestrial environments. We, therefore, suggest that there should be a link between the history of duplications and losses in these protein families and that of habitat changes within the phylum. Using a broader sampling of tardigrade genomes, we have identified new sequences (74 CAHS, 8 MAHS, 29 SAHS, 22 EtAHS alpha, 18 EtAHS beta, and 21 MRE11 sequences) from 8 tardigrade families (Batillipedidae, Halechiniscidae, Echiniscidae, Milnesiidae, Macrobiotidae, Murrayidae, Hypsibiidae, and Ramazzottiidae) across 13 genera (*Batillipes*, *Echiniscus*, *Viridiscus*, *Cornechiniscus*, *Halechiniscus*, *Milnesium*, *Paramacrobiotus*, *Macrobiotus*, *Mesobiotus*, *Dactylobiotus*, *Ramazzottius*, *Hypsibius*, and *Acutuncus*) in both Eutardigrada and Heterotardigrada (fig. 1). Additionally, we identified 30 fatty acid–binding protein sequences from across these 13 genera to act as an outgroup for our analyses of SAHS, alongside previously published fatty acid–binding proteins. We constructed phylogenetic analyses for each anhydrobiosis-associated protein and mapped the history of these families on Fleming and Arakawa (2021)'s 18S/28S tardigrade phylogeny, to better understand the evolutionary history of anhydrobiosis in Tardigrada.

## Results and Discussion

### Revising CAHS Nomenclature and Determining Orthology

CAHS proteins are the largest family of heat-soluble proteins considered in this study, and additionally possess the largest number of sub-families. Although the behaviors of some of these proteins have been characterized (Yagi-Utsumi et al. 2021; Malki et al. 2022), their phylogenetic relationships to one another have not. As such, CAHS proteins are named in order of their discovery; for example, *Hypsibius* CAHS1 may not share a common ancestor with *Ramazzottius* CAHS1 under the current nomenclature. Although localized to the Eutardigrada, CAHS shows great variation, with some forming granules in response to desiccative stress and others forming filaments within the cell (Yagi-Utsumi et al. 2021; Malki et al. 2022).

**Fig. 2.**
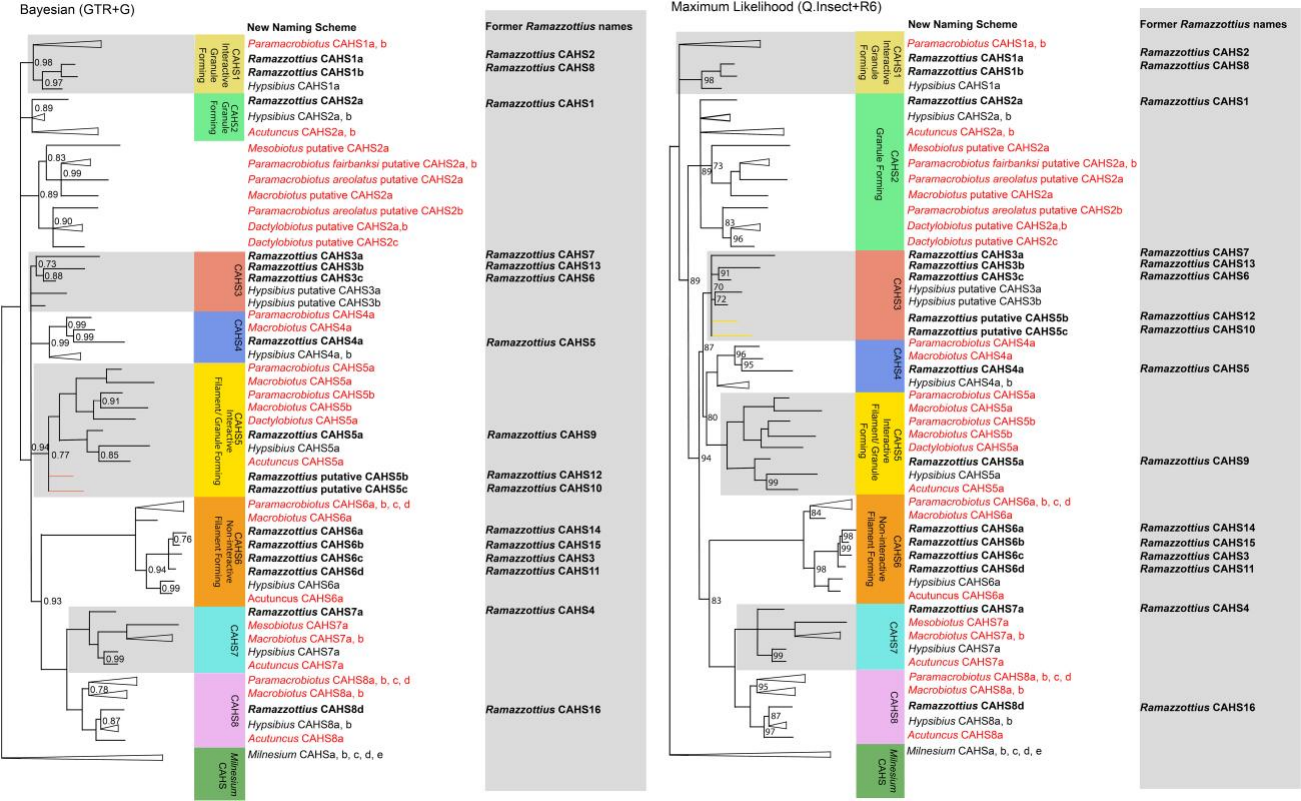
Bayesian consensus and maximum likelihood phylogenies depicting the CAHS protein family within Tardigrada. The former names of the *Ramazzottius* CAHS proteins are depicted to the right of each *Ramazzottius* CAHS protein, alongside the phylogenetically informative nomenclature for the CAHS protein introduced within this paper. The sequences introduced in this study are colored in red, and the sequences recovered from prior studies are in black. PPs <1 are marked on the Bayesian phylogeny, and PPs <0.7 are reduced to polytomies. UBS values <100 are marked on the maximum likelihood phylogeny, and nodes with <70 support are reduced to polytomies. The nodes comprising only independent duplications have been collapsed for readability, unless these independent duplications belong to *Ramazzottius*, where they have been retained for clarity in nomenclature revision. The conflicts between the Bayesian and the maximum likelihood phylogeny have been highlighted by coloring the branches of the corresponding group in the opposite phylogeny. The assumed ancestral functional annotations have been added to the group where sequences have been functionally characterized, but individual variation is also present within groups: *Hypsibius* CAHS2*b*, for example, forms fibrous gels but is a member of the granule-forming group.

Here (fig. 2), we introduce a new naming scheme for CAHS proteins based on homology groupings. In this naming scheme, the numbers correspond to each of the eutardigrade shared CAHS homologs, and later duplications are designated with a letter following the homology group numeral. The final letter in each designation does not indicate homology—*Hypsibius* CAHS 1a and *Ramazzottius* CAHS1a are not homologous, but rather serves to indicate the number of copies from this subfamily present in the genus.

In cases where we were unable to recover a representative of a given CAHS group in the Macrobiotidae, Ramazzottiidae, or Hypsibiidae, we interpreted this as an indicator of loss of the CAHS homolog. However, because of low BUSCO completeness, in the case of *Dactylobiotus* (supplementary table S1, Supplementary Material online), we do not interpret the absence of CAHS homologs as representative of losses in Murrayidae.

### CAHS in *Milnesium*

We were able to locate five CAHS sequences in *Milnesium tardigradum* from an assembly previously uploaded to GenBank (see Supplementary material online, Bioproject PRJNA34121) and as such rooted the phylogeny taxonomically on *Milnesium*. We did this as no outgroup sequences outside Eutardigrada were available, and the most closely related gene family to CAHS is unknown (fig. 2). These sequences were recovered as a monophyletic group, suggesting an independent duplication of CAHS within *Milnesium*. Additionally, this implies that the shared ancestor of the Eutardigrada possessed only a single CAHS sequence. All but one of the recovered *Milnesium* CAHS sequences were recovered in the third quartile of normalized taxon-specific Relative Composition Frequency Variability (ntRCFV) values for the dataset (supplementary table S2, Supplementary Material online), suggesting that they are more compositionally heterogeneous than the majority of CAHS sequences (Fleming and Struck 2023). However, all but one is recovered within 2 standard deviations (SDs) of the whole dataset's normalized Relative Composition Frequency Variability (nRCFV) value, which suggests that they are not significant outliers when compared with the background heterogeneity of the dataset.

### CAHS in Parachela: Interactive Granule-Forming CAHS1 and Granule-Forming CAHS 2

In contrast to Apochela, within the Parachela, the evolutionary history of CAHS appears to be far more complex. Our phylogenetic analysis (fig. 2) shows that CAHS can be divided into at least 8 distinct groups. Each of these groups contains representatives from both Hypsibiidae and Macrobiotidae, suggesting that the last common ancestor of these two families—and by extension the last common ancestor of all Parachela with the exception of Isohypsibiidae, which did not appear in our study (Guil et al. 2018; Fleming 2023)—possessed at least one copy of each of these eight CAHS groups. In some cases, these groups are not consistent with the species phylogeny—where this is the case, our reasoning is clarified in the relevant section (fig. 1).

The history of the earliest divergences within CAHS is unclear with regard to our initial phylogeny. CAHS2 comprises sequences from Hypsibiidae and Ramazzottiidae. In *Hypsibius*, it shows two independent duplications, resulting in three paralogs labeled 2a, b, and c, respectively. In *Acutuncus*, one independent duplication can be observed. CAHS1, meanwhile, comprises sequences from Hypsibiidae, Ramazzottiidae, and Macrobiotidae. The lack of sequences found in both *Acutuncus* and *Macrobiotus* in this group, despite CAHS1 being found in *Hypsibius* and *Paramacrobiotus*, implies an independent loss of CAHS1 in both *Acutuncus* and *Macrobiotus*. Meanwhile, both *Paramacrobiotus* and *Ramazzottius* appear to have each undergone a single independent duplication of CAHS1 (fig. 2). Furthermore, the nature of the earliest diverging CAHS group is unclear—our maximum likelihood topology favors CAHS1 but cannot recover them as a monophyletic group (fig. 2), whereas our Bayesian analysis recovers a monophyletic CAHS1 in a polytomy with CAHS2.

Notably, both CAHS1 and 2 have members that have been functionally characterized: *Ramazzottius* CAHS2a (previously known as *Ramazzottius* CAHS1) and *Hypsibius* CAHS1a (previously known as CAHS8). Both of these proteins have been identified as “granule forming” (Yagi-Utsumi et al. 2021; Malki et al. 2022). As both are found within early diverging clades in the tree, it suggests that granule-forming behavior may be the ancestral mode of the protein. Studies of *Hypsibius* CAHS2b, however, have shown that it forms fibrous gels, indicating that there may be variations in behavior across the group (Tanaka et al. 2022).

Alongside CAHS1 and 2, a third monophyletic group of sequences can be found in this early diverging polytomy, separated from the remainder of CAHS. These sequences have not been attributed to any CAHS family, and in our study, they were found only in Macrobiotid and *Dactylobiotus* samples. As such, they may represent the members of CAHS2, which according to our maximum likelihood analysis (fig. 2), would otherwise have been independently lost in the shared ancestor of Macrobiotidae and Murrayidae. However, our Bayesian analysis recovers them in a polytomy (fig. 2), in which case they may represent a separate CAHS family that was independently lost in the shared ancestor of Hypsibiidae and Ramazzottiidae. This group has been labeled “putative” CAHS2, as their relationship with CAHS1 and 2 is unclear. Although further sampling will be needed to clarify the relationships of these proteins, the internal structure of this group within our phylogeny merits further discussion. One subclade contains three representatives from *Dactylobiotus* and one sequence from *Paramacrobiotus*. The three sequences from *Dactylobiotus* are arranged together, evidencing two lineage-specific duplications. The lack of Macrobiotid sequences beyond *Paramacrobiotus* in this subclade, however, is notable and suggests that the placement of the *Paramacrobiotus* sequence in this clade is artifactual. The alternative would be that the gene coding for this protein was independently lost in other members of the Macrobiotidae, which would imply multiple, lineage-specific losses—a less parsimonious scenario.

The other subclade, meanwhile, contains no samples from *Dactylobiotus*, but instead comprises a single sequence from *Macrobiotus* and *Mesobiotus* and two sequences from *Paramacrobiotus*. The latter two sequences are found in a sister relationship, suggesting an independent duplication of CAHS in *Paramacrobiotus* in this subclade.

### CAHS in Parachela: Uncharacterized CAHS3

CAHS3, 4, and 5 are found in a polytomy alongside all other CAHS sequences, as sisters to the previously discussed groups (fig. 2). CAHS3 is not a monophyletic group within our Bayesian analysis (fig. 2), comprising three groups in a polytomy. However, it is monophyletic in our maximum likelihood analysis (see Supplementary material online). Here, it is recovered with moderate ultrafast bootstrap support (UBS) for the node connecting *Hypsibius* CAHS3a and 3b (UBS = 81.6) and very low UBS for the node connecting these two *Hypsibius* sequences with *Ramazzottius* CAHS3a, b, and c (UBS = 57.7; formerly *Ramazzottius* CAHS7, 13, and 6, respectively). In our maximum likelihood analysis, CAHS3 is recovered in a clade sister to CAHS 4 and 5 (fig. 2).

### CAHS in Parachela: CAHS4 and Interactive Filament/Granule-Forming CAHS5

CAHS4 presents an internal topology worthy of further examination. The group comprises two *Hypsibius* sequences and one sequence from each of *Ramazzottius*, *Macrobiotus*, and *Paramacrobiotus* (fig. 2). The two *Hypsibius* CAHS4 sequences appear to be the result of a single duplication. However, *Ramazzottius* CAHS4a (formerly *Ramazzottius* CAHS5) is recovered as a sister to *Macrobiotus* CAHS4a. In turn, this clade is recovered as a sister to *Paramacrobiotus* CAHS4a. This suggests the possibility of three CAHS4 paralogs in the eutardigrade common ancestor, with independent losses in the CAHS4 subclade represented by *Hypsibius* sequences in Ramazzottiidae and Macrobiotidae, independent losses in the CAHS4 subclade represented by the lone *Paramacrobiotus* sequence in Hypsibiidae and Ramazzottiidae, and a further independent loss of the *Macrobiotus* + *Ramazzottius* CAHS4 clade in the Hypsibiidae. However, considering the data paucity for this clade, which comprises only five orthologs, it is highly possible that this is a topological artifact despite the high posterior probability (PP) at each node.

CAHS5 is the third of the four CAHS groups that contain a member that has been functionally characterized—*Ramazzottius* putative CAHS5b (formerly known as *Ramazzottius* CAHS12; Tanaka et al. 2022). Putative CAHS5b is an interactive filament/granule-forming CAHS, and so it is interesting that, with this intermediate behavior, it is recovered as part of an intermediate phylogenetic clade (alongside CAHS3 and 4) between the characterized granule-forming clades (CAHS1 and 2) and the filament-forming group (CAHS6). Putative CAHS5b and c appear as early diverging sequences in a polytomy at the origin of the clade. This may indicate a *Ramazzottius*-specific CAHS9 that has been independently lost in all other Tardigrada studied or a long-branch attraction artifact. We consider the latter to be more likely, as in our maximum likelihood topology, these sequences are recovered as members of CAHS3 (fig. 2). We note these sequences as potentially problematic sequences to be subject to further analysis in the future but have named them in line with our Bayesian analysis. These sequences are thus labeled “putative CAHS5b and c,” allowing them to be easily removed should later analyses prove them to belong to another CAHS group.

Following putative CAHS5b and c, the CAHS5 group then diverges into a Hypsibiidae + Ramazzottiidae subclade (comprising sequences from *Hypsibius*, *Acutuncus*, and *Ramazzottius*, but showing no indication of independent duplication) and a *Dactylobiotus* + *Paramacrobiotus* subclade. Within the latter subclade, one group contains sequences from *Paramacrobiotus* and *Macrobiotus*, whereas another comprises sequences from *Paramacrobiotus*, *Macrobiotus*, and *Dactylobiotus*. This subclade shows no signs of independent duplication, and therefore, the multiple CAHS5 present in Macrobiotidae may represent a shared duplication event in the common ancestor of Macrobiotidae and Murrayidae following the split from the ancestors of Hypsibiidae and Ramazzottiidae (fig. 1).

### CAHS in Parachela: Noninteractive Filament-Forming CAHS6 and Uncharacterized CAHS7 and CAHS8

CAHS6 is the final of the four functionally characterized CAHS groups recovered in this study. Here, three independent duplications in both *Paramacrobiotus* and *Ramazzottius* were identified, and the sequences within the group were arranged consistently with the species phylogeny (figs. 1 and 2). CAHS6a (formerly CAHS3; Tanaka et al. 2022) was found to form noninteractive filaments. Considering the well-resolved nature of the clade—particularly in comparison with other CAHS groups—characterizing the behavior of these additional orthologs of CAHS6—*Rammazzottius* CAHS6b, 6c, and 6d (formerly 15, 14, and 11)—may help us understand whether these functional behaviors are truly phylogenetically conserved.

CAHS7 is a monophyletic group comprising sequences from *Ramazzottius*, *Mesobiotus*, *Macrobiotus*, *Hypsibius*, and *Acutuncus*. Two sequences found in *Macrobiotus* suggest an independent duplication in the clade, whereas the *Ramazzottius* sequence (formerly CAHS4), found as a sister to all other CAHS7 sequences, suggests the possibility of a duplication in CAHS7 in the shared parachelid ancestor that was independently lost in all clades but the Ramazzottiidae. It is, however, more likely that this is a topological artifact, especially as no *Ramazzottius* CAHS sequences were recovered in the CAHS7 subclade that contains all other CAHS7 sequences.

CAHS8 comprises sequences from *Paramacrobiotus*, *Macrobiotus*, *Ramazzottius*, *Hypsibius*, and *Acutuncus*. These sequences produce a topology that is consistent with the species phylogeny. A single independent duplication can be observed in both *Macrobiotus* and *Hypsibius*, whereas three independent duplications are found in *Paramacrobiotus*.

Overall, we recovered 12 CAHS sequences from *Hypsibius*, 6 from *Acutuncus*, 16 from *Ramazzottius*, 9 from *Macrobiotus*, 16 from *Paramacrobiotus*, 4 from *Dactylobiotus*, and 5 from *Milnesium*. Although there is clearly a high propensity for independent duplications in the clade, these heat-soluble proteins appear to group into monophyletic clades, which suggests that multiple copies of CAHS predate the divergence of Parachela. In addition, the large number of CAHS paralogs recovered from *Paramacrobiotus* suggests that this genus might be incredibly important in cryptobiotic studies as a comparative organism to *Ramazzottius*. It does, however, additionally highlight the paucity of data within Eutardigrada: further sampling from the Murrayidae and the addition of high-quality samples from Richtersiidae and Isohypsibiidae would greatly aid our understanding of the evolution of CAHS. The new nomenclature put forward within this study will hopefully further ease communication between researchers regarding CAHS—especially considering the potential for phylogenetically conserved behavior within CAHS clades—as we further characterize tardigrade biology at a more granular level (Yagi-Utsumi et al. 2021; Malki et al. 2022; Tanaka et al. 2022).

### The Phylogenetic Relationships of MAHS

MAHS is a novel mitochondrial protein family found exclusively within tardigrades (Tanaka et al. 2015). It is expressed in this organelle in contrast to CAHS (which can be found in the cytoplasm) or SAHS (which can be found in the extracellular space and secretory organelles), where it appears to play a role similar to that of Late Embryogenesis Abundant proteins during anhydrobiosis (Tanaka et al. 2015).

**Fig. 3.**
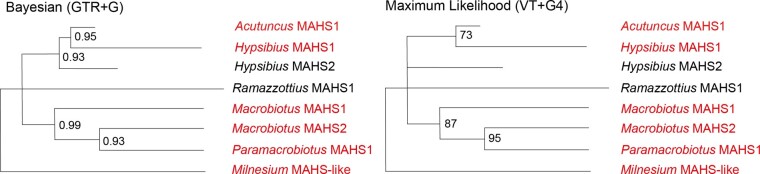
Bayesian consensus and maximum likelihood phylogenies depicting the MAHS protein family within Tardigrada. The sequences introduced in this study are colored in red, and the sequences recovered from prior studies are in black. PPs <1 are marked on the Bayesian phylogeny, and PPs <0.7 are reduced to polytomies. UBS values <100 are marked on the maximum likelihood phylogeny, and nodes with <70 support are reduced to polytomies.

The single MAHS-like sequence that was identified from our three separate *Milnesium* samples is recovered as a separate branch in the unrooted tree (fig. 3), and therefore, the MAHS phylogeny (fig. 3) can be parsimoniously rooted upon the Milnesiidae. The resultant phylogeny suggests a history that is congruent with the species phylogeny.

Within Hypsibiidae, we found two MAHS sequences in *Hypsibius* and one in *Acutuncus* (fig. 3). *Hypsibius* MAHS1 was located as a sister to *Acutuncus* MAHS1, whereas *Hypsibius* MAHS2 was recovered as a sister to this group. This implies a shared duplication event prior to the divergence of *Hypsibius* and *Acutuncus*, followed by the loss of the second copy of MAHS in *Acutuncus*.

Within the Macrobiotidae, a similar pattern was observed (fig. 3). We found two MAHS sequences in *Macrobiotus* and one in *Paramacrobiotus*. *Macrobiotus* MAHS1 was located as a sister to *Paramacrobiotus* MAHS1, whereas *Macrobiotus* MAHS2 was recovered as a sister to this group. As in the Hypsibiidae, this implies a shared duplication event prior to the divergence of *Macrobiotus* and *Paramacrobiotus*, followed by the loss of the second copy of MAHS in *Paramacrobiotus*.

In our Bayesian analyses (Supplementary material online), a sister group association between the Hypsibiidae and the Macrobiotidae MAHS sequences was preferred (with low PP = 0.59; Supplementary material online), whereas in our preliminary maximum likelihood analyses, the Hypsibiidae + Ramazzottiidae was preferred, although with low (UBS = 50; Supplementary material online). As such, we consider that the low probability topology in our Bayesian analysis may be attributed to a long-branch attraction artifact, potentially resulting from the inclusion of the *Milnesium* MAHS sequence in the phylogeny. Unfortunately, as removing *Milnesium* would result in the creation of a three-taxon tree problem, we cannot further test this assertion.

### MAHS-Like Sequences in *Milnesium*

In agreement with prior studies on MAHS (Tanaka et al. 2015), no representatives of this family were located outside of Eutardigrada (fig. 3). However, this study contains the first MAHS sequences discovered within the Macrobiotidae as well as the first MAHS-like sequences in Milnesiidae. The sequence recovered from *Milnesium* lacks the important functional mitochondria targeting–peptide seen in “true” MAHS sequences (see Supplementary material online for multiple sequence alignments; Tanaka et al. 2015), and therefore, establishing it as functionally similar to the MAHS sequences recovered from other eutardigrade families would be erroneous. However, we were unable to locate any MAHS or MAHS-like sequences in our arthrotardigrade or heterotardigrade samples. This expands our knowledge of MAHS to members of both the apochelids and the parachelids, and following our review of genomes outside of Eutardigrada, we suggest that the MAHS-like protein was acquired in the common ancestor of Apochela and Parachela.

### Revising SAHS Nomenclature

SAHS is a third eutardigrade-specific heat-soluble protein family, found predominantly within the extracellular space and secretory organelles. Although these sequences have been characterized in a preliminary analysis into two phylogenetic subfamilies based on shared molecular characteristics, their phylogenetic relationship with the fatty acid–binding proteins that have long been hypothesized to be the sister group to SAHS has never been tested (Yoshida et al. 2017; Miyazawa et al. 2022). Furthermore, within the current subfamily characterization, one comprises only sequences found in *Ramazzottius* (Yoshida et al. 2017), which further highlights both poor sampling in Tardigrada and a lack of understanding of the development of heat-soluble proteins in the phylum.

**Fig. 4.**
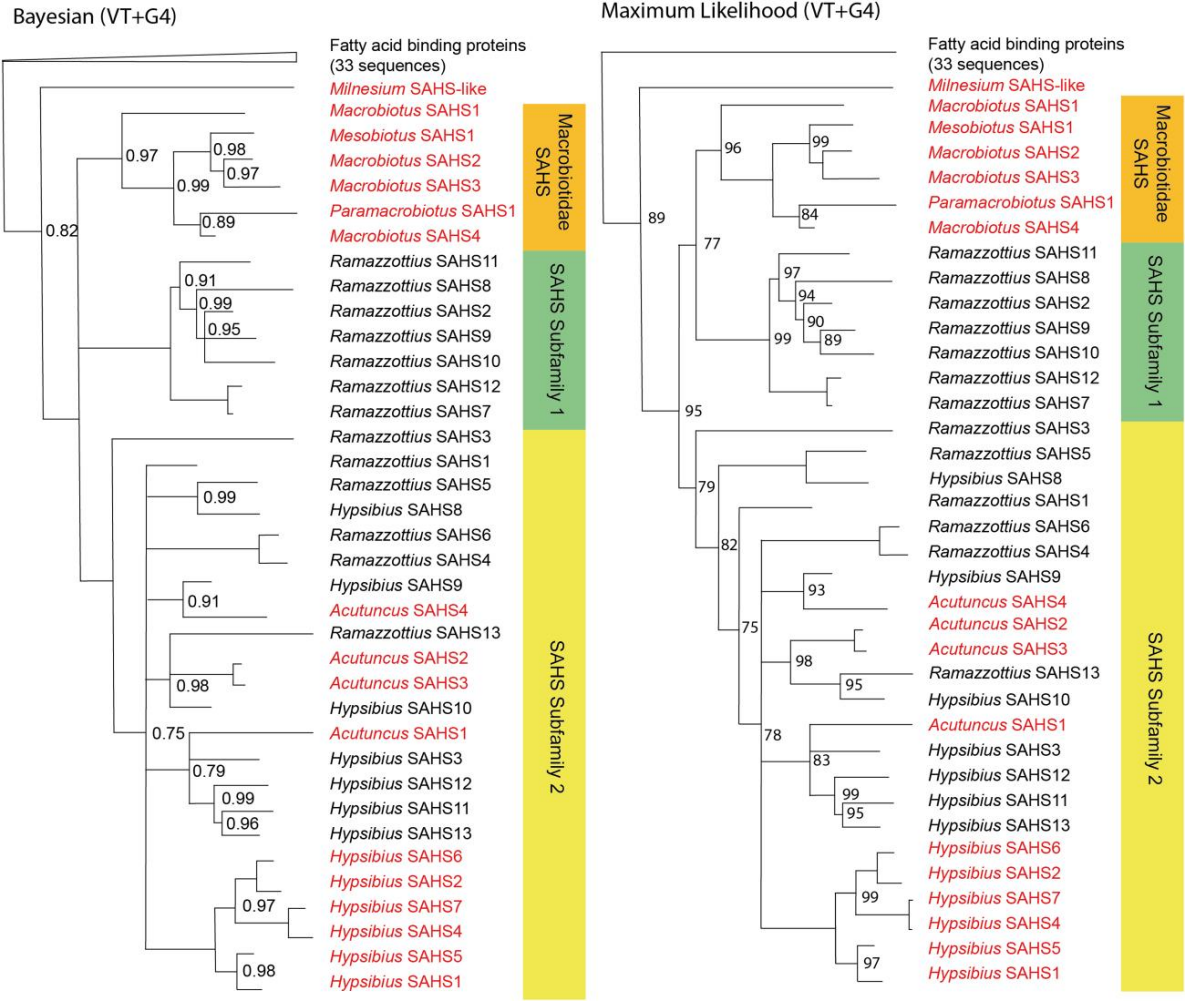
Bayesian consensus and maximum likelihood phylogenies depicting the SAHS protein family within Tardigrada. The sequences introduced in this study are colored in red, and the sequences recovered from prior studies are in black. The SAHS subfamilies recovered by Yoshida et al. (2017) are depicted on the right, alongside the new Macrobiotid SAHS. PPs <1 are marked on the Bayesian phylogeny, and PPs <0.7 are reduced to polytomies. UBS values <100 are marked on the maximum likelihood phylogeny, and nodes with <70 support are reduced to polytomies. As SAHS retains the old nomenclature, which is not consistent with their phylogenetic relationships, the clades comprising only independent duplications have not been collapsed.

Our own Basic Local Alignment Search Tool (BLAST) analyses also suggested a structural and potentially phylogenetic affinity of SAHS with the fatty acid–binding proteins located elsewhere within Metazoa (Fukuda and Inoue 2018; fig. 4). Starting from a dataset consisting of SAHS and SAHS-like sequences identified from Macrobiotidae, Hypsibiidae, and Milnesiidae, alongside an outgroup of fatty acid–binding proteins, we found that, although SAHS resolves as a clear monophyletic group, internal resolution within SAHS was poor under both maximum likelihood and Bayesian methodologies (fig. 3; Supplementary material online). We additionally recovered the two previously proposed SAHS subfamilies (fig. 4; Yoshida et al. 2017). This includes SAHS subfamily 1, as in Yoshida et al (2017), comprising only *Ramazzottius* sequences. However, SAHS subfamily two was revealed to be much more complex (fig. 4; for further discussion, see below). As such, unlike our CAHS analysis, we have elected not to revise the naming scheme of *Ramazzottius* SAHS. We suggest that, although the family needs a nomenclature revision, a new naming scheme at this point is unlikely to be robust to future increases in sampling, and by extension, would only cause further confusion.

### The Phylogenetic Relationships of SAHS

The single *Milnesium* SAHS-like sequence resolved as a sister to all other SAHS sequences (fig. 4). As with MAHS, we refer to this *Milnesium* sequence as SAHS-like rather than as a “true” member of SAHS. SAHS is expressed in the tardigrade-specific storage cells; however, no such expression of SAHS has yet been found in *Milnesium* (Tanaka et al. 2023), and without the confirmation of expression in these cells, we have chosen to err on the side of caution when assigning nomenclature.

Within the parachelan SAHS sequences, the first node branches into a polytomy of three monophyletic groups (fig. 4). As such, we discuss these groups in reading order from the top of figure 4. The first of these groups comprises four sequences from *Macrobiotus*, one sequence from *Paramacrobiotus*, and one sequence from *Mesobiotus*. However, the four *Macrobiotus* SAHS are divided between three separate clades. One is recovered as a lone sequence, one as a sister to *Paramacrobiotus* SAHS1, and two *Macrobiotus* SAHS are recovered together, as a sister to *Mesobiotus* SAHS1. This suggests that two shared duplications occurred at the base of the Macrobiotidae and that the lack of the *Mesobiotus* and *Paramacrobiotus* SAHS sequences in these subclades is either a result of independent loss or insufficient sampling. In our Bayesian analysis, this clade comprises all Macrobiotid SAHS sequences recovered in this study, and it appears that further duplications were shared only among the Hypsibiidae + Ramazzottiidae.

The second of the three groups comprises *Ramazzottius* SAHS2, 7, 8, 9, 10, 11, and 12, suggesting six independent duplications inside this clade (fig. 4). This group is referred to as SAHS subfamily 1, as recovered in Yoshida et al (2017). Whether these Ramazzottiid sequences are sister to the Macrobiotid SAHS clade is unclear, although this association is recovered both in our maximum likelihood analysis (fig. 4) and in our Bayesian analyses, although with low support (0.4; see Supplementary material online). This suggests the possibility of independent losses in the Hypsibiidae in this earliest diverging group of SAHS, but further sampling will be required to clarify this polytomy.

The third group comprises all the sequences previously identified as belonging to SAHS subfamily 2 (Yoshida et al. 2017) and all of our new *Hypsibius* and Acutuncus SAHS sequences. It begins with an early diverging lone *Ramazzottius* SAHS sequence, SAHS3 (fig. 4). This implies either a long-branch attraction artifact drawing this sequence to the base of this group or an independent loss of *Ramazzottius* SAHS3 analog in all other Hypsibiidae + Ramazzottiidae considered in this study. We consider the former to be more likely considering the lower PP of the immediate descendant node (PP = 0.75) and the less robust ultrafast bootstrap score of the node itself in our maximum likelihood analysis (UBS = 79; fig. 4). In our maximum likelihood analysis, the early diverging *Ramazzottius* SAHS3 is followed by a group comprising *Ramazzottius* SAHS5 and *Hypsibius* SAHS3 and then by the lone diverging *Ramazzottius* SAHS1. This is either a further indication of frequent independent losses in SAHS or, more likely, an evidence of insufficient sampling.

In our Bayesian analysis, the aforementioned lower PP node, however, is a polytomy comprising seven monophyletic groups and comprising all the sequences previously classified as members of SAHS subfamily 1. As such, we discuss each of the monophyletic groups within the polytomy in order of increasing number of sequences, as depicted in figure 4. Only one of these seven clades is a single-sequence clade—*Ramazzottius* SAHS1. Three two-sequence clades exist, one comprising *Ramazzottius* SAHS5 and *Hypsibius* SAHS8, another comprising *Hypsibius* SAHS9 and *Acutuncus* SAHS4, and a third comprising *Ramazzottius* SAHS6 and 4—the result of an independent duplication.

One four-sequence clade comprises *Ramazzottius* SAHS13, *Hypsibius* SAHS10, and *Acutuncus* SAHS2 and 3. As *Acutuncus* SAHS2 and 3 are recovered as sisters to each other, it indicates an independent duplication of this SAHS in *Acutuncus* (fig. 4).

One five-sequence clade comprises *Acutuncus* SAHS1 and *Hypsibius* SAHS3, 11, 12, and 13. This clade has one subclade comprising *Hypsibius* SAHS11, 12, and 3, which suggests two independent duplications at this point in the tree in *Hypsibius*. Depending on the resolution of the internal polytomy within this five-sequence clade, there may be a third independent duplication: *Hypsibius* SAHS13 is either a sister to *Acutuncus* SAHS1, which would imply an independent loss of the SAHS3/11/12 homolog in *Acutuncus*, or a sister to 3/11/12, which would imply a further independent duplication in *Hypsibius*.

Finally, the largest clade is also the simplest in its evolutionary history. This group comprises only six *Hypsibius* SAHS sequences—SAHS1, 2, 4, 5, 6, and 7—indicating at least five further independent duplications in *Hypsibius*.

### Further SAHS-Like Sequences in *Milnesium*

We recovered three SAHS-like sequences from the same *Milnesium* sample. However, two of these samples were discarded due to compositional heterogeneity (see Materials and Methods). Considering the long-branched nature of the *Milnesium* SAHS-like that was retained in our study, their true identity may not be SAHS or SAHS-like (Supplementary material online). However, there remains the possibility that they represent true additional paralogs of SAHS within *Milnesium*, and considering the independent duplications of SAHS within the other families of Tardigrada, this assertion cannot be discounted.

### The Phylogenetic Relationships of MRE11

MRE11 is the only protein family in this study not restricted to Tardigrada. MRE11 is an important protein involved in the DNA damage response (Stracker and Petrini 2011), and as such, is critically important to recovery from cryptobiosis (Hashimoto et al. 2016). Considering this, understanding whether multiple copies of MRE11, which is a phenomenon rarely found outside Tardigrada (Hashimoto et al. 2016), are the result of a single, shared duplication event or the result of multiple independent duplication events may be critical to understanding the history of cryptobiotic recovery in the clade, as well as provide a comparative basis with respect to other metazoan phyla.

**Fig. 5.**
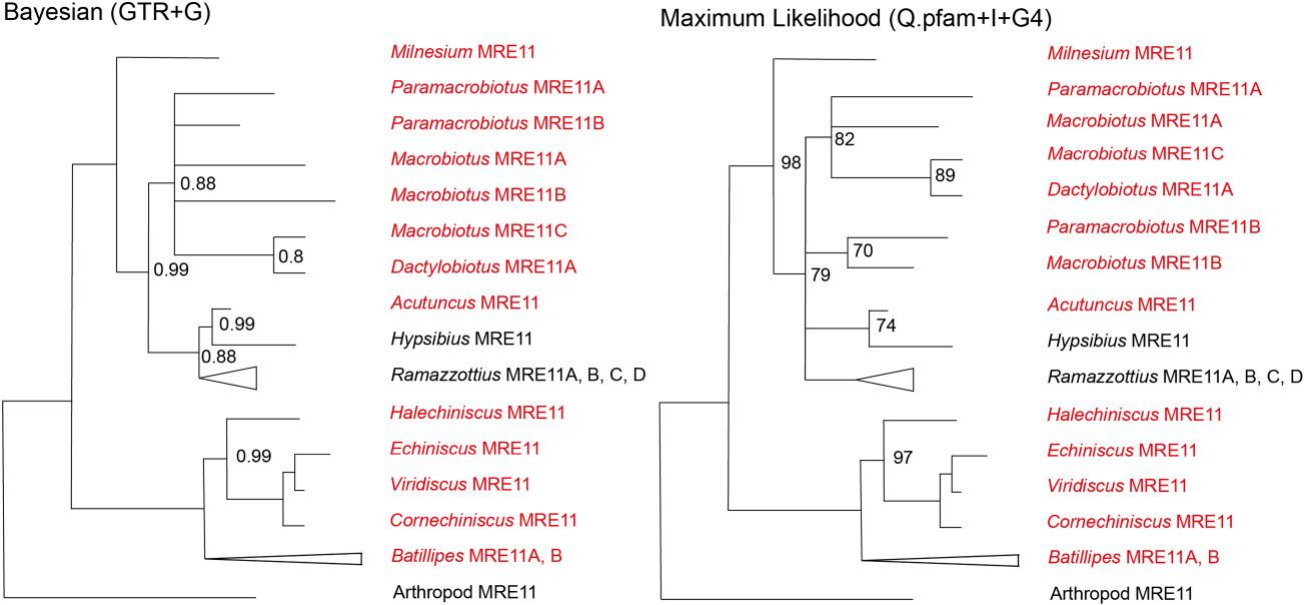
Bayesian consensus and maximum likelihood phylogenies depicting the MRE11 protein family within Tardigrada. The sequences introduced in this study are colored in red, and the sequences recovered from prior studies are in black. PPs <1 are marked on the Bayesian phylogeny, and PPs <0.7 are reduced to polytomies. UBS values <100 are marked on the maximum likelihood phylogeny, and nodes with <70 support are reduced to polytomies. The nodes comprising only independent duplications have been collapsed for readability.

MRE11 shows the simplest pattern of duplication of the studied desiccation-resistant protein families (fig. 5). One copy of MRE11 appears to be shared across the phylum. An independent duplication event has occurred within Arthrotardigrada, potentially purely within *Batillipes*.

Within Eutardigrada, Macrobiotid and Murrayid sequences are recovered in a large polytomy with the exception of *Macrobiotus* MRE11C and *Dactylobiotus* MRE11A. This suggests a shared Macrobiotid/Murrayid MRE11 duplication. In addition, it indicates either a further Macrobiotidae-specific MRE11 duplication or two separate independent duplications of MRE11 in *Paramacrobiotus* and *Macrobiotus*, respectively. A Hypsibiidae + Ramazzottiidae clade can also be found as a sister to the Macrobiotid + Murrayid clade. Here, *Hypsibius* and *Acutuncus* show no duplications of MRE11, but *Ramazzottius* appears to have undergone three independent duplications of MRE11 (fig. 5).

### Exploring EtAHS Alpha and EtAHS Beta

Although CAHS, SAHS, and MAHS appear to be eutardigrade specific and MRE11 is found across Metazoa, EtAHS has been reported in only one heterotardigrade species so far: *E. testudo* (Murai et al. 2021). These proteins bear many similarities to CAHS: both appear to share a similar nuclear magnetic resonance profile and also form an alpha-helix in response to water. However, at the amino acid level, CAHS and EtAHS bear no resemblance to each other, and therefore, it is likely that this represents an independent evolution of desiccation resistance within the group. Furthermore, EtAHS alpha and beta are not paralogous proteins and are not related to each other. The breadth of these groups, however, has not yet been tested—EtAHS may be *Echiniscus* specific, Echiniscoidea specific, or more broadly specific to Heterotardigrada.

Because of their similar distribution patterns and the limited nature of prior sampling, we address these two protein families together. Our phylogenetic analysis showed that not only were both EtAHS alpha and beta restricted to the Echiniscoidea—with no sequences being found in *Batillipes*—but also that the protein was prone to independent duplication. All four of the Echiniscoid study species exist in terrestrial environments, and all four minimally possess one copy each of EtAHS alpha and beta. Although there may be variations in how different species interact with EtAHS, it suggests that, minimally, only two copies of EtAHS alpha and one of EtAHS beta are sufficient to regulate the anhydrobiotic response, but that more copies of these sequences may provide additional benefits (Murai et al. 2021).

### The Phylogenetic Relationships of EtAHS Alpha and Beta

**Fig. 6.**
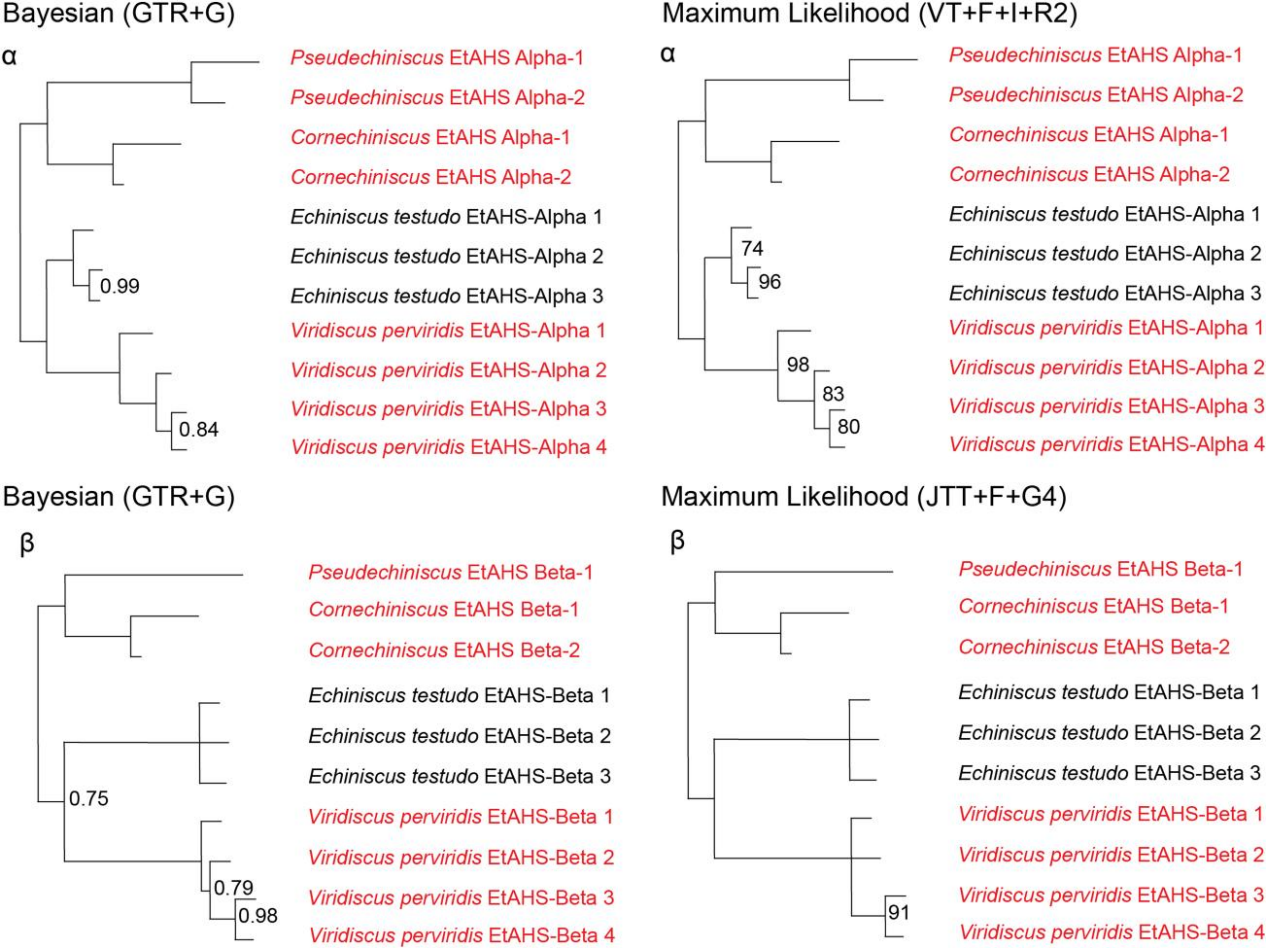
Bayesian consensus and maximum likelihood phylogenies depicting the EtAHS alpha (panel α) and beta (panel β) protein families within Tardigrada. The sequences introduced in this study are colored in red, and the sequences recovered from prior studies are in black. PPs <1 are marked on the Bayesian phylogeny, and PPs <0.7 are reduced to polytomies. UBS values <100 are marked on the maximum likelihood phylogeny, and nodes with <70 support are reduced to polytomies. As all observed duplications in this family are independent, the nodes comprising only independent duplications have not been collapsed.

The shared ancestors of *E. testudo* and *Viridiscus perviridis* appear to have possessed only one EtAHS alpha, but through independent duplication, *V. perviridis* possesses four copies, whereas *E. testudo* possesses three copies (fig. 6, panel α). Outside of *Echiniscus* and *Viridiscus*, species-specific duplication also appears to have occurred within *Cornechiniscus* and *Pseudechiniscus*. *Cornechiniscus* possesses two copies of EtAHS alpha, whereas *Pseudechiniscus* possesses two copies of EtAHS alpha (fig. 6, panel α).

As in EtAHS alpha, *E. testudo* and *V. perviridis*, the shared ancestors of *E. testudo* and *V. perviridis* appear to have possessed only one EtAHS beta. In another stark similarity between the two families, *E. testudo* now independently possesses three copies, whereas *V. perviridis* possesses four copies through independent duplication (fig. 6, panel β). Outside of *Echiniscus* and *Viridiscus*, a single species-specific duplication has occurred within *Cornechiniscus*, but only one copy of EtAHS beta could be found in *Pseudechiniscus*.

### EtAHS Alpha and Beta Consensus Sequences

For EtAHS alpha and beta, as the first demonstration of the distribution of these families outside of *E. testudo*, we generated consensus sequences for both alignments. The EtAHS alpha consensus sequence was derived as


$$\begin{array}{c} FYNN\{RG\}TY\{IT\}\{FY\}\{MLT\}LE\{LV\}PC\{DE\}AYL\{GST\}\\ \{GQP\}\{RA\}G\{GV\}\;\;\; \end{array}$$

This 25 amino acid region starts at *E. testudo* EtAHS alpha 1 position 45 and continues until position 70, spanning 4% of the total multiple sequence alignment and 7% of the reference sequence.

Meanwhile, the EtAHS beta consensus sequence was derived as


$$\begin{array}{c} \begin{array}{c} DGVK\{QKL\}PIDLTRVLA\{IRN\}TP\{DE\}VLE\{QK\}\{IV\}\{DN\}\{DV\}\\ V\{FY\}FFFPN\left\{KG\}Q\{IS\}GR\{IV\}EFE\{HQ\}GQ\{PA\}\{NED\}EL\{EA\right\}\\ RITCR\{PA\}PCRGGP\{RSK\}\left\{NSTQ\}\{AT\right\} \end{array} \end{array}$$

This 65 amino acid region appears to be highly conserved over the 21 identified EtAHS beta sequences. This suggests that EtAHS beta is a highly conservative protein, as this region spans a full 15% of the overall EtAHS beta alignment and 20% of the reference sequence. It starts at EtAHS beta 1 position 140 and continues until position 205.

We suggest that these consensus sequences may prove useful in identifying EtAHS alpha and beta in the future, although they are not aware of the histological properties that might cause such high conservation in these regions of the sequences (Murai et al. 2021).

### The Origins of Terrestrialization in Tardigrada as Inferred from Heat-Soluble Protein Family Evolution

**Fig. 7.**
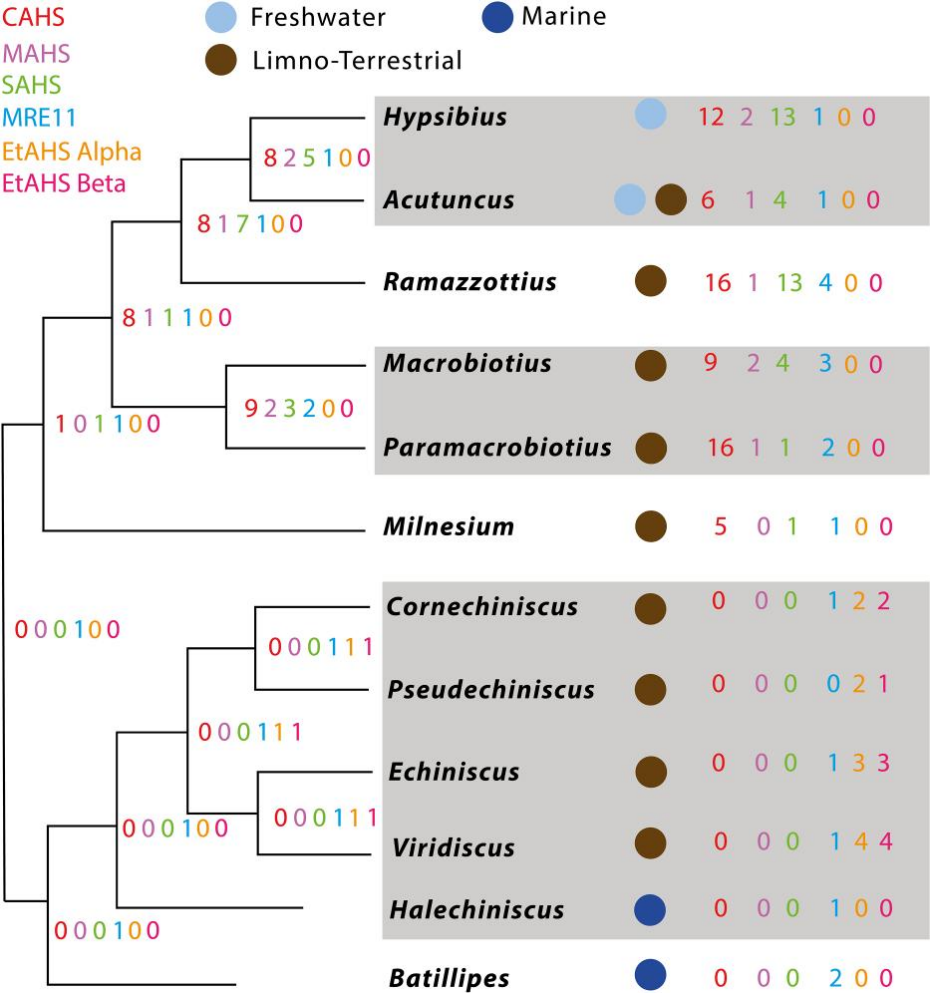
An overview of the numbers of tardigrade heat-soluble protein orthologs across the phylum. CAHS is represented in blue, MAHS in purple, SAHS in green, EtAHS alpha in red, EtAHS beta in orange, and MRE11 in black. The habitat of each sampled tardigrade genus is indicated as circles on the leaves: marine environments are in dark blue, freshwater in light blue, and limnoterrestrial in brown.

Two constants are visible across all the tardigrade species and protein families covered within this study. The first is that both independent duplications and independent loss are common. Much of the diversity of CAHS, SAHS, and MAHS in Eutardigrada appears to be the result of independent duplications, many of which are not even shared at the order level. The second is that multiple copies of the genes coding for these proteins are not necessarily more common within strongly anhydrobiotic species. *Hypsibius exemplaris* is a notable example (Arakawa 2022; fig. 7), because, although the members of this genus can be found in both limnoterrestrial and freshwater habitats, *H. exemplaris* has thus far been found only in freshwater (Gąsiorek et al. 2018; Poprawa et al. 2022). Bearing this in mind, we can only weakly use the history of duplications and losses to assess the likely ecologies of extinct, ancestral tardigrades.

Our heat-soluble protein family analysis suggests that although stem Eutardigrada may have been marine, transition to a more desiccation-prone environment—either freshwater or limnoterrestrial—had potentially already occurred in the common ancestors of Apochela and Parachela, as one copy of each of the heat-soluble protein families (CAHS, SAHS, and MAHS) is shared across both clades. In the event of a more ancient transition to the limnoterrestrial environment, we would have expected to find CAHS, SAHS, MAHS, or EtAHS alpha and beta shared between Eutardigrada and Heterotardigrada. Our inability to find CAHS, SAHS, and MAHS in Heterotardigrada corroborates prior studies on the group (Murai et al. 2021).

Within the Echiniscoidea, our results similarly suggest that one copy each of EtAHS alpha and beta may well be sufficient to regulate the anhydrobiotic response (Murai et al. 2021; fig. 6). Most notably for the broader history of terrestrialization within the Tardigrada, the development of a wholly separate mechanism for desiccation response, and the lack of CAHS, SAHS, and MAHS proteins in Heterotardigrada, implies that the Echiniscoidea terrestrialized independently of the other tardigrade groups. These conclusions are corroborated by our results within the Eutardigrada, which, when assessed with regard to the species phylogeny, implies that the shared Heterotardigrade–Eutardigrade ancestor was marine (Fleming and Arakawa 2021; figs. 6 and 7).

## Materials and Methods

### Gene Identification

We sequenced and identified new genes in 36 new genomes and transcriptomes (supplementary table S1, Supplementary Material online) using BLAST (e-value = 10e−10) against a set of known sequences of the candidate family (CAHS, SAHS, MAHS, MRE11, EtAHS alpha, EtAHS beta, and fatty acid–binding proteins) obtained from the NCBI. Following this, the sequences of significant similarity were subjected to a secondary BLAST search against the whole nonredundant protein database (nr), with only those that scored the highest match (of e-value ≥10e−10) against a previously identified candidate family member retained. New tardigrade genomes were sequenced and assembled following the protocols outlined in Arakawa et al. (2016), and the relevant sequences used in this analysis are included in the Supplementary material online alongside their NCBI accession numbers. Both previously published sequences and new sequences were used to construct the phylogenies later in the study.

Further gene searches were attempted in *Milnesium* to determine whether more divergent SAHS and MAHS genes were present in the genus. Here, HMM profile searches were employed using all other SAHS and MAHS genes as the profile dataset, and BLAST searches were also undertaken using the EDSSMAT62 matrix rather than BLOSUM62, which is more sensitive to comparisons of intrinsically disordered proteins such as SAHS and MAHS (Trivedi and Nagarajaram 2019). This search retained only sequences over 80 amino acids with e-values >1 that did not score the highest match against another protein family when compared with the nr dataset using a BLAST search employing a BLOSUM62 scoring matrix.

### Alignment and Phylogenetic Tree Construction

Newly identified members of the candidate families were combined with the previously identified sequences and aligned using MUSCLE (Edgar 2004). Preliminary trees were constructed in IQTree (Nguyen et al. 2015). CAHS, MAHS, MRE11 EtAHS alpha, and EtAHS beta phylogenetic trees were constructed using the GTR + G model and SAHS using the VT + G4 Model in PhyloBayes 4.1 (in the latter case as recommended by ModelFinder following poor tree resolution under GTR + G—see Supplementary material online; Lartillot and Philippe 2004, 2006; Lartillot et al. 2007; Kalyaanamoorthy et al. 2017). Convergence was assessed by comparing the maximum discrepancies observed over the bipartitions and effective sample size in bpcomp and tracecomp. For all analyses, two independent chains were run. A burnin of 50% of the sample size was used for all analyses, sampling every 50th tree following the burnin period. UBS and PP >80/0.8 were considered to show a reasonably confident level of robustness and support for the topology, and support below 70/0.7 was collapsed to polytomies in the relevant figures.

Because of a paucity of sampling and data quality (supplementary table S1, Supplementary Material online), we identified and discussed the homologs of all target protein families at the genus, rather than species, level. When a single sequence was recovered from a monophyletic group with single sequences from other samples of the same genus, these sequences were classified as the same target protein. If multiple sequences were recovered from the same sample in a monophyletic group to the exclusion of sequences belonging to members outside the genus, it was considered an independent duplication.

Furthermore, we were conservative in our inference of independent duplications to avoid overestimating gene duplications due to the presence of poor coverage genomes in our dataset (supplementary table S1, Supplementary Material online; Ko et al. 2022). Although all sequences recovered from assemblies were included in our phylogenetic analysis, where coverage was below 40×, we undertook an independent BLAST analysis comparing all sequences belonging to that gene family recovered from the same assembly. In cases where the percentage sequence identity was >98%, it was not inferred as an independent duplication in our Results and Discussion section (see figs. 2–7; supplementary table S1, Supplementary Material online). It was relevant only for our analysis of CAHS, where 15 *Paramacrobiotus* sequences were identified as potentially representing only 7 CAHS homologs.

In the case of SAHS, a suitable outgroup was found in the fatty acid–binding protein family, to which it bears structural resemblance and molecular similarity (Fukuda and Inoue 2018). However, for the other tardigrade-specific candidate families (EtAHS alpha, EtAHS beta, MAHS, and CAHS), no suitable outgroups could be found. In this case, these trees are presented in this manuscript, with roots displaying the most parsimonious number of duplications. For the case of the candidate family not specific to the Tardigrada, MRE11, this tree was rooted with an outgroup composed of nontardigrade MRE11 sequences.

As the *Milnesium* SAHS and MAHS-like sequences appeared to diverge significantly from the SAHS and MAHS sequences present in other Eutardigrades, the presence of rogue sequences and long-branch attraction was a considerable concern. As such, each candidate *Milnesium* SAHS and MAHS-like sequence was assessed independently by measuring the normalized relative compositional frequency values of the dataset, and candidate sequences with a ntRCFV >3 SDs from the whole dataset's nRCFV were considered to be significantly compositionally heterogeneous (supplementary table S2, Supplementary Material online). nRCFV scores were calculated using RCFV_Reader (Fleming and Struck 2023), available on GitHub: https://github.com/JFFleming/RCFV_Reader/.

### Consensus Sequence Identification

Consensus sequences for EtAHS alpha and beta were identified using ConSeq (Berezin et al. 2004). The longest uninterrupted consensus phrase—defined as no more than one consecutive “X,” representing any amino acid—was then extracted from the consensus sequence as provided by ConSeq, and any alternative amino acids within the consensus sequence were then added.

## Supplementary Material

evad217_Supplementary_Data

## Data Availability

All new sequences identified by this study have been uploaded to Genbank, with accession numbers provided in supplementary table S1, Supplementary Material online, alongside notes of whether sequences were synonimized due to low coverage, as per our Materials and Methods. Alignments and newick tree files are made available as Supplementary material online. BUSCO assembly scores and coverage depth, alongside the locality in which the specimens were found and relevant publication notes, can be found in supplementary table S1, Supplementary Material online.
