# Correction to: The Evolution of Temperature and Desiccation-Related Protein Families in Tardigrada Reveals a Complex Acquisition of Extremotolerance

**DOI:** 10.1093/gbe/evae043

**Published:** 2024-03-27

This is a correction to: James F Fleming, Davide Pisani, Kazuharu Arakawa, The Evolution of Temperature and Desiccation-Related Protein Families in Tardigrada Reveals a Complex Acquisition of Extremotolerance, *Genome Biology and Evolution*, Volume 16, Issue 1, January 2024, https://doi.org/10.1093/gbe/evad217)

In the original publication of this manuscript, there was an error in the colouring for *Halechiniscus* which should be coloured as a marine, not terrestrial species. Figure one should read:

**Figure:**
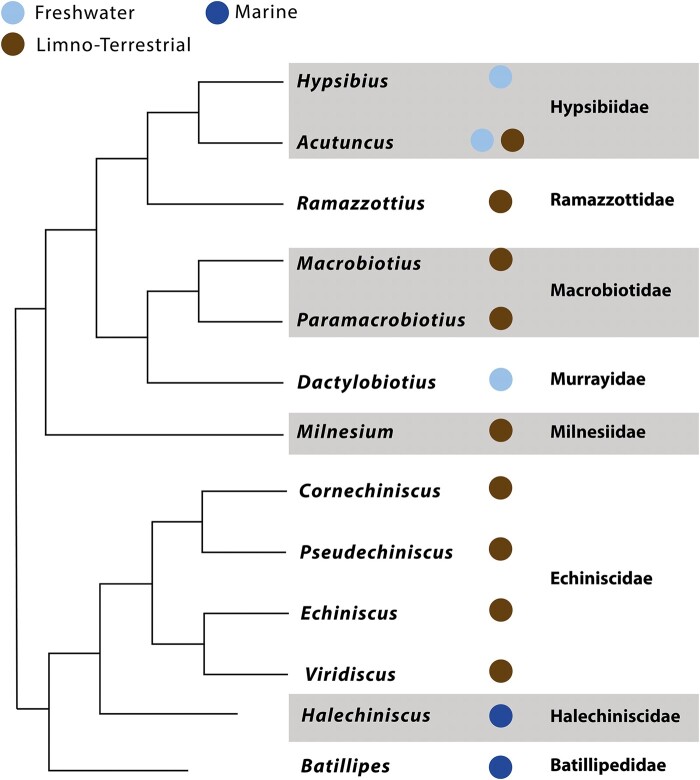


instead of:

**Figure:**
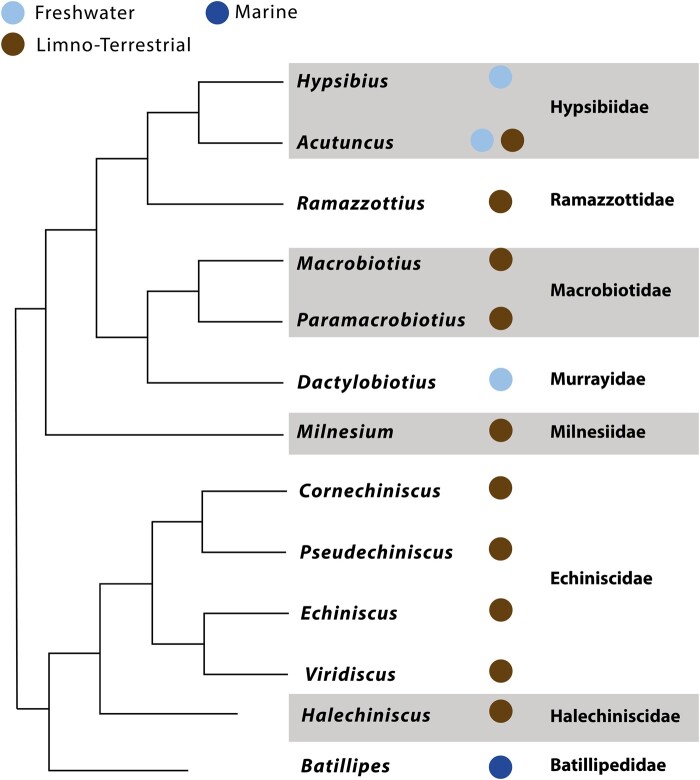


Figure 7 should read:

**Figure:**
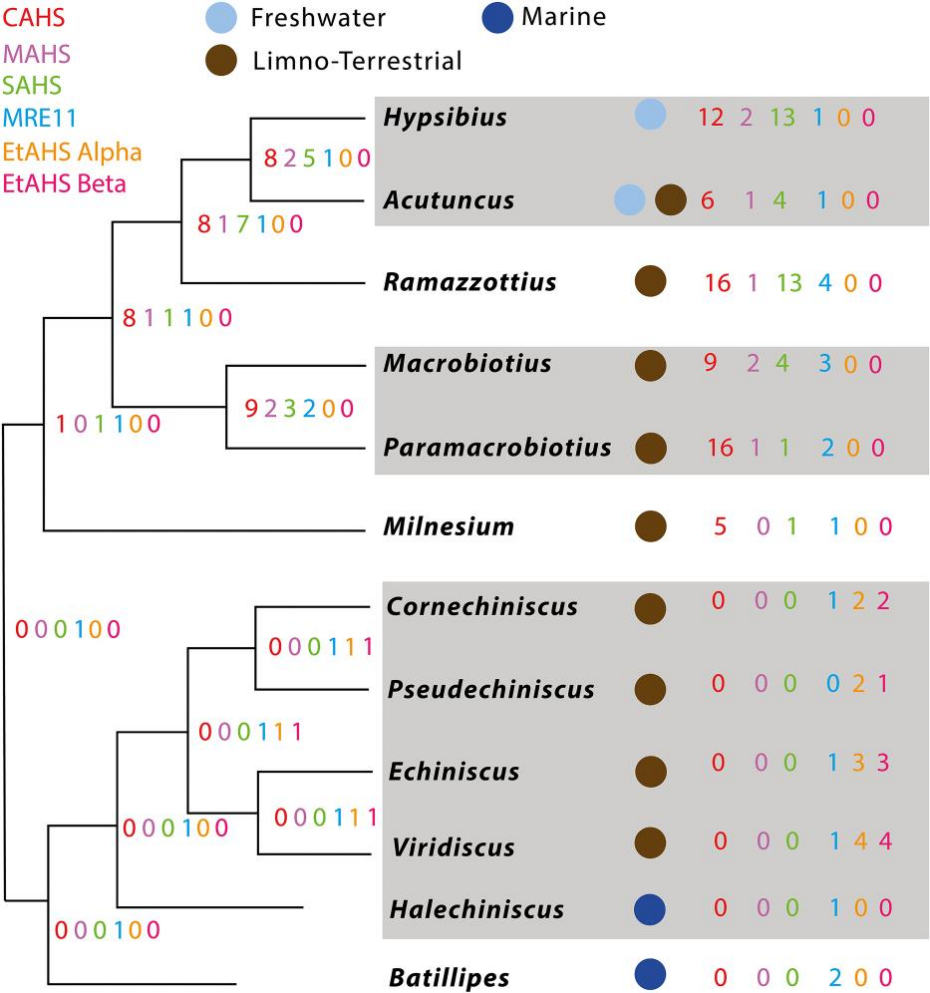


instead of:

**Figure:**
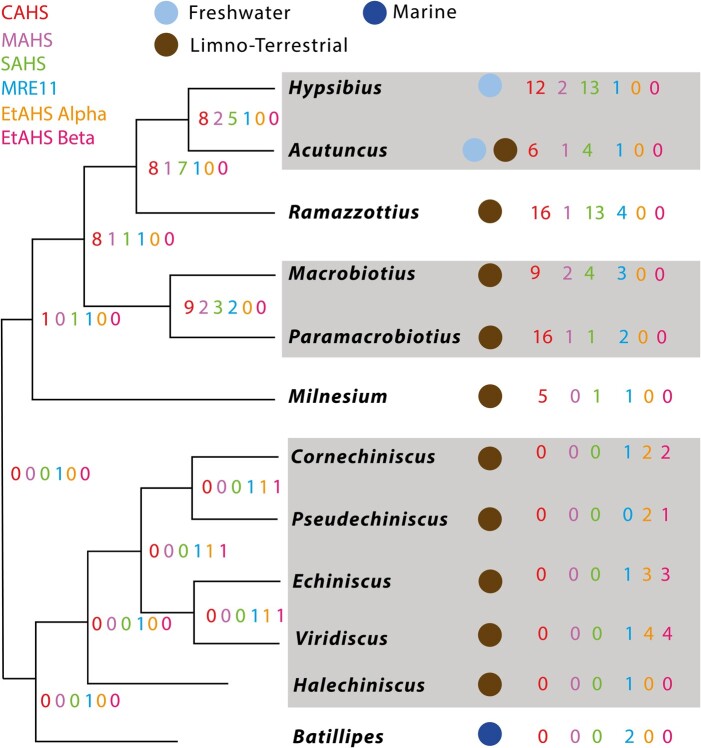


Table S3 was missing from the supplementary data: this has now been added to the paper's Supplementary Data section.

These emendations have been made to the article.

