# Correction to: The Evolution of Temperature and Desiccation-Related Protein Families in Tardigrada Reveals a Complex Acquisition of Extremotolerance

**DOI:** 10.1093/gbe/evaf018

**Published:** 2025-02-17

This is a correction to: James F Fleming, Davide Pisani, Kazuharu Arakawa, The Evolution of Temperature and Desiccation-Related Protein Families in Tardigrada Reveals a Complex Acquisition of Extremotolerance, *Genome Biology and Evolution*, Volume 16, Issue 1, January 2024, https://doi.org/10.1093/gbe/evad217

In the originally published online version of this manuscript, details in the caption to Figure 7 were errored.

These should read: “[…]CAHS is represented in **red**, MAHS in purple, SAHS in green, EtAHS alpha in **yellow**, EtAHS beta in **pink**, and MRE11 in **blue**.[…]” instead of: “[…] CAHS is represented in **blue**, MAHS in purple, SAHS in green, EtAHS alpha in **red**, EtAHS beta in **orange**, and MRE11 in **black**.[…]”.

These errors are outlined only in this correction notice to preserve the version of record.

